# Disability History Gets to Work

**DOI:** 10.1017/S0960777325101148

**Published:** 2025-08-29

**Authors:** Coreen Anne McGuire

**Affiliations:** Department of History, Faculty of Arts and Humanities, Durham University, Durham, UK

In the infamous second episode of the fictional American comedy TV show *The Office*, Steve Carell’s gratingly clueless character Michael encourages his staff to wear index cards with particular races on them for ‘diversity day’ training. I was reminded of this while reading about the real experience of blind psychologist Adrienne Asch at a ‘group dynamics’ programme in the mid-1980s during which ‘participants were asked to stand under a sign representing their group identification’.^1^ Asch was the only disabled person at the meeting, and so, reluctantly, moved away from the sign that said ‘woman’ and stood under the sign that read ‘disabled’. When another participant approvingly told her that she was right to stand there and not ‘deny’ her identity, Ach exclaimed ‘It’s for people like you that I have to stand under that sign. You and your attitudes put me there, not my blindness itself ‘.^2^ Asch (who had a PhD in social psychology from Columbia University) went on to work as an advocate for women and disabled people in the field of bioethics. This moment, when Asch articulated the social model of disability to defend herself as both disabled and a medical professional, is replicated many times with several other individual professionals in Hogan’s richly detailed account of how various medical fields interacted with disability activism in the late twentieth century.

The book shows how US medical professionals in three fields (clinical psychology, paediatrics and genetic counselling) responded to disability advocacy in the post-war period. Importantly, it demonstrates how professionals with lived experience of disability became self-advocates while working within medicine. These examples of social model activism taking place within the medical model powerfully undercut the social/medical binary framework, though they also demonstrate how powerfully it has functioned as a political tool. It was of course as a political tool that the medical/social model framework originally developed, to separate out impairment from disability in the way that Asch did, making it clear that it was the structures of an inaccessible society that disabled people (the social model) and not their individual bodies (the medical model). From its origins in British Marxist politics, this way of thinking inspired a generation of historians to consider the disabling impacts of society in different periods of history, largely concentrating on the modern period. However, the false dichotomy inherent to social/medical model thinking has meant that disability history has developed divergently, even in opposition to, medical history.^3^ In this way, Hogan’s book can be usefully read alongside calls to integrate disability history with histories of medicine and science, such as the 2024 *Osiris* special issue on this topic.^4^

Hogan has previously called for more nuance in thinking about how medical historians and historical subjects working within medicine have upheld (or rather, not upheld) the medical model.^5^ And he has now demonstrated how rigorous empirical work can reveal examples of supportive and productive dialogues between disabled advocates and clinicians. Fulsomely detailed and supported by extensive archival research and interviews, it is in these ‘disability dialogues’ – a phenomenon coined by one of the disability advocates Hogan investigates to refer to disabled individuals forcefully engaging with the medical profession – that the book’s power is fully evident. The oral histories with self-advocates contain fascinating and revealing accounts of how disabled people worked to change perspectives on disability within medicine. For instance, self-advocate Marsha Saxton engaged with genetic counsellors to alleviate disability discrimination and recounts a genetic counselling session in which the counsellor told her ‘If I had known “spina bifadas” could turn out as well as you, then I would not have recommended selective abortion’.^6^ This (horrifying) conversation exemplifies how personal relationships between advocates and professionals encouraged deeper awareness of more optimistic understandings of disabled individuals.

Hogan further explains that clinicians’ reluctant responses to more ‘positive, accepting, and sociopolitical perspectives on disability’ were not attributed to the medical model but rather linked to concerns about their work, ‘concerns about professional role, identity, and prestige’.^7^ This point is perhaps too nuanced. After all, these clinicians were concerned about their identity and role *as medical* professionals and attempted to furrow their disciplines within the contours of the *medical* field. In fact, this book in many ways convinced me of the ongoing utility of the medical model for understanding the extent of historical ableism within medicine as, though there were many examples of supportive and productive dialogues between disabled advocates and clinicians, there were also many examples of extreme and ongoing ableism within the various institutions discussed. Indeed, though this is a book of many individuals, it is also a book of many institutions, which means the reader must parse multiple and varied acronyms.

Attempts to keep track of the acronyms comes to a head in chapter five, which is devoted to the efforts to create subspecialisations of developmental disabilities, largely spearheaded by Arnold Capute. His campaign to develop neurodevelopmental disabilities into a recognised paediatric sub-speciality was clearly advanced for his own professional advancement, with little to do with care and interest in disabled children, who he discussed repeatedly in relation to the aphorism ‘you can’t make a race horse out of a pig’.^8^ Further, he suggested that children with autism should be described as ‘communicative perverts’ so he could label them under the CP (cerebral palsy) umbrella in service of his broader ambition and institution building.^9^ This chapter is a good example of the enduring importance of the medical model and its continued explanatory utility. However, the book also helps to complicate and expand disability history historiography by bringing a welcome focus to intellectual disability and disability within the family. Disability history’s initial focus on recovering accounts of historical individuals’ independent disability activism has resulted in an understandable reluctance to focus on interdependence and reliance. However, David Turner and Daniel Blackie have recently demonstrated how attending to the ‘complex family relations and economic responsibilities of impaired working people’ can allow us to tell new stories about disabled activism, revealing, for instance, its important role within the factory reform movement.^10^ The book’s focus on post-war paediatrics and genetic counselling means that relational disability – as it is experienced within the family – is often at the forefront of this volume, and sometimes disability advocates were parents or siblings. This motivation, for instance, spurred on the efforts of Robert Cooke and Eunice Kennedy Shriver to fund university-affiliated clinical facilities for training in mental retardation and to stop the strategic withholding of treatment from newborn disabled children. Cooke’s two daughters had significant developmental disabilities resulting from a rare chromosomal disorder (cri du chat syndrome) while Eunice’s brothers John, Robert and Ted Kennedy were influenced by the experiences of their sister Rosemary, who had been institutionalised following ‘the catastrophic effects of a lobotomy’.^11^ Hogan describes how ‘For some, a surprise encounter with disability – in their patients, their own children, or the wider community – was a defining moment, when they suddenly realized how poorly prepared and ill-informed they were about how to respond and help’.^12^ It is in the recounting of these moments (these disability dialogues) that the book is most compelling. It tells a story not of one individual but of many individuals and in doing so reveals how small moments and chance encounters shape lives and, subsequently, professions and disciplines.

Discipline shaping is also of key concern to Esme Cleall, whose edited collection, *Global Histories of Disability, 1700–2015: Power, Place and People*, aims to demonstrate the importance of considering disability history alongside global history. This is a significant contribution, given the field’s overwhelming focus on sources from the United States and the United Kingdom and on western understandings of disablement. Making this move, she explains, brings out the important links between both disability and colonialism and between disability and race. In the introduction to the collection, Cleall outlines the Anglo-American bias of the historiography and explicates the surprising ‘failure of disability history to have much coverage in the Global South despite the fact that physical disability is often caused by phenomena that, in the twenty-first century, are more commonly experienced there, such as warfare, poverty and famine’.^13^ Like Hogan’s book, Cleall’s collection is not underpinned by a medical/social model framework and the key focus of the volume is instead on how imperialism has shaped what it has meant to be disabled. Notably, Cleall pleas for historicism and warns against theoretical accounts of disability, warning historians not to theorise about disability without grounding ‘theorising about disability in the empirical’.^14^

To do so she has collected eight case studies that demonstrate that the ‘more global examples we have of the experience and construction of disability, the better we can understand the praxis and theory of what it means to be disabled’.^15^ Indeed, the lack of embedded perspectives in non-western contexts is a significant issue in the field. One of the reasons why disability history (and indeed all history) is important is because it shows that the way things are now was not inevitable, that things were different in the past and could be again in the future. Change happens in time but also *in space* – and this is one vital contribution of this volume – it shows how conceptions of disability differ from place to place and even differ in the same place under different regimes, as in Sam De Schutter’s chapter on Kenya pre- and post-independence and Gildas Brégain’s valuable chapter on the administration of disability policy in the French colonies. Brégain’s chapter is unusual in that it is a comparative case study of more than one territory, a methodology that Blackie and Moncrieff have identified as lacking within disability historiography.^16^ By analysing the differing levels of care and compensation offered to disabled civilians in Algeria, Madagascar and the French West African Federation, Brégain demonstrates ethnic and geographic inequalities in the way disabled people were treated across the French empire. Brégain’s investigation of the shaping of disability through colonialism specifically overlaps with the focus of just over half of the chapters collected, while the others consider how imperialism (broadly defined) has created disablement. As a result, institutional and colonial sources are heavily used throughout, but the methodologies used to access these sources differ and result in the voices of the disabled coming through more strongly in some chapters than in others. As in Hogan’s book, moments of biographical relief can help temper the effect of difficult and distant institutional sources.

Iain Hutchinson’s chapter, for instance, is a micro-history of the life of a Scottish man (William Bailie) who became mentally unwell while working for the East India Company in India in 1813. It was critical for the EIC that their representatives projected an image of capability and strength and so visibly disabled workers were quickly hidden. William, who had converted to Islam, was aware of his illness and sought out privacy so that he could hide it and attempt to remain well with access to fresh air and quiet seclusion back in Scotland. The chapter explores William and his close family member’s understanding of his symptoms but also illuminates how the ‘effects of the sun on Europeans in India and other hot climates taxed the enquiring minds of colonial medical practitioners’.^17^ On one level, this chapter demonstrates how elite colonial control and ‘carefully manicured images of power, control and superiority were challenged by the onset of mental ill health’.^18^ However, on another level it more simply presents us with a moving portrait of a man who rejected categorisation as disabled and continually ‘strove to assert agency over his residual able-bodiedness’.^19^ Caroline Lieffers’s chapter similarly elevates the voices and actions of the disabled subjects she explores in her accounting of the logic of ableism ‘as evinced in representations of artificial limbs and Indigenous people in the continental West’.^20^ The voices and actions of Joseph La Flesche, Ceca Yamni and a third (unnamed) indigenous man who engaged with artificial prosthetics and what Lieffers terms the ‘violence of American cure’ are vividly mapped out in this innovative exploration of imperial ableism. By excavating the accounts of indigenous men who experimented with prosthetic limbs, Lieffers also explores how ‘racialised assessments’ of indigenous people’s capacities resulted in them routinely being designated as ‘incapable’.^21^

That disability has, in this way, been attributed generally to non-white people as a way of justifying ongoing oppression is demonstrated most strongly in chapters by Stefanie Hunt-Kennedy and Madhwi. Madhwi’s chapter is also a comparative study, connecting the experiences of indentured labourers on plantations in the British colonies of Mauritius and Natal. In Natal, workers were categorised as ‘able-bodied’ or ‘non–able-bodied’ and paid according to this classification (non–able-bodied men were paid the same as women). In Mauritius all were made to perform the same tasks, meaning sickness became common. In both places, illness and disability were punished through wage and ration deductions. The most significant finding to arise from this chapter is that the brutality of the indenture system meant that some emigrants disabled themselves to mobilise against the plantation economy. Madhwi interprets this as an act of resistance and agency, explaining that ‘some emigrants chose to become disabled as a means to oppose the indentured system, allowing them to deny being able-bodied and contest their position as part of the work force. Disability was an opportunity to develop “voice”‘.^22^ This interpretation will be of especial interest to emerging scholars working on disability in relation to unpaid labour, as it shows the complex ways in which disability can be linked to a kind of power, ‘agency within the plantation economy’.^23^ This was not by any means an easy way out; people had to invalid themselves in extreme ways and, if these methods failed, often ‘opted for suicide’.^24^

Whether self-inflicted or not, the disabling impact of work cuts across almost all these chapters but is theorised in different ways. The industrialisation thesis, the idea that the advent of modern capitalism in the nineteenth century ‘created’ disability by making apparent that non-standard bodies were unsuitable for the workforce, is reproduced uncritically in Beckman’s chapter but subverted in Hunt-Kennedy’s chapter on slavery and in Madhwi’s work on indentured servants.^25^ In her chapter on eighteenth-century slavery-induced disability in the Caribbean (a summary of her book on the topic), Hunt-Kennedy explains that disability was ‘pervasive among the enslaved populations of the Caribbean, and it shaped their daily lives and interactions with each other and with slaveowners’.^26^ Far from disability removing labourers from the workforce, the workforce was both conceptually and literally disabled by design – they became more disabled the longer they remained at work. Wearing out the body through work was the point of slavery, which means disability was both inescapable and persistent. Efforts to rehabilitate to create a more productive labour force were a feature only of waged labour forces.

Sam De Schutter explains this in a more nuanced articulation of the industrialisation thesis by focusing on Kenya before and after independence. De Schutter builds on both Julie Livingstone and Esme Cleall’s work to provide a case study that demonstrates ‘the colonial invention of disability’.^27^ His focus on institutions and policies aimed at ‘turning disabled people into productive workers’ covers rehabilitation and training both within and outwith institutions.^28^ The rehabilitation centre discussed initially focused on ex-servicemen – Africans who had fought for the British Empire and had been treated poorly after the First World War – so that in the 1940s there was a sense that this failure needed to be made up for through rehabilitation as well as pensions. But the main reason for its development was to create a more productive labour force. De Schutter extends Sarah Rose’s historicising of the industrialisation thesis to similarly argue that disability did become understood more narrowly in relation to productivity, as ‘Disability was thus solely defined in terms of whether one could work or not, and if so, what type of work he (and this citizen was always gendered masculine in the discourse) could perform’.^29^ He emphasises the links between the British project in East Africa and legislation developed for disabled people in Britain, as in both cases full citizenship was linked to participation in the workforce, though ‘for colonial subjects, that participation was not linked to citizenship and was much more of a duty than a right’.^30^ Even after independence, De Schutter stresses that focus on productivity remained paramount as the colonial definition of disability as dependence continued in policies designed to make disabled individuals contribute to the economy.^31^

The relationship between disability and colonialism and disability and race are made most explicit in Hunt-Kennedy’s book, *Between Fitness and Death: Disability and Slavery in the Caribbean*, which shows how theorising black bodies as inherently deviant justified their enslavement.^32^ It is hard to overstate just how impressive an achievement *Between Fitness and Death* is as a piece of scholarly work. The scope and scale of its research is great; chapter four alone uses over one thousand primary sources. Despite its size and level of detail, it is easy to read in the sense that the writing is pacey and engaging – though its exploration of the unceasing brutality of the slave system is challenging. The methodology Hunt-Kennedy brings to bear on these sources is impressive too; she reads sources created by slave owners creatively and sensitively, and further maintains that ‘Wherever possible I draw on works written by former captives during the abolitionist period, all the while recognising that even these sources bear the imprint of white abolitionists’.^33^

She uses these sources to connect disability as critical to enslavement in three ways. First, it was by discussing African bodies as disabled that they could be presented as a different race, justifying their subsequent bondage. This puts disability at the very centre of race in a way that makes it seem astounding that it has not previously been considered central by historians of slavery. Second, slavery produced disablement in a variety of ways, through the brutal wearing down of the mind and body, through literal dismemberment and violence and through what Hunt-Kennedy terms ‘diseases of unfreedom’. These diseases of unfreedom include diseases resulting from malnutrition, unsanitary cramped living conditions and the withholding of medical treatment. However, disability was also produced directly, as slave owners routinely dismembered their workers through work or punishment (cutting off feet for running away, for example) and marked their bodies with scars from flogging and other punishments. Third, legislation disabled the enslaved socially and politically by restricting their autonomy and freedom of movement. The legislative example that struck me most forcefully was the 1702 law that compensated whites who became disabled while tracking down runaway slaves ‘as a testament to their service’, meaning British legislation in Jamaica compensated for disability before any compensation for disability existed for workers in Britain.^34^ Indeed, Hunt-Kennedy directly challenges the industrialisation thesis and the understanding of disability that the field has been built on, explaining that the ‘prevalence of impairment, disfigurement, and deformity on Barbadian and Jamaican sugar plantations shows that the colonial Caribbean challenges traditional Eurocentric timelines of disability history, which maintain that disability in its modern sense emerged with the onset of industrialization in nineteenth-century Europe and North America’.^35^ In this way, *Between Fitness and Death* works, like Cleall’s book, to challenge the Eurocentric biases of disability history while also challenging its chronological partiality to the modern period. Its framing of race as fundamentally conceptualised through disability is an important recognition that should spur on more work in this vein in different places and periods.

However, what Hunt-Kennedy means by lawmakers imposing ‘metaphoric disabilities’ is that freedom of movement was curtailed metaphorically as well as forcefully through slave codes and ticketing systems that worked ‘to imagine and construct enslaved blacks as wholly dependent and limited cripples’.^36^ This is a definition of disability that does not particularly involve the body or mind of a person and as such, it raises metaphysical questions about what disability is that historians ought to consider carefully. In the valuable work to extend disability history and use concepts from disability studies to bear on the past, some scholars have used an expanded and somewhat blurred definition of disability. Disability is not something that can be strictly defined; it is a mutable and contextual experience, and part of the point of disability history is to make that clear by showing how it has been understood differently in the past. That said, we need to categorise it for the concept to remain meaningful and to allow us to accurately name other forms of intersecting oppressions – like racism, like poverty, like classism, like sexism, like caste-based violence. These different forms of oppression may well be disabling, but they do not by themselves constitute disability. Conceptual clarity is vital for historical work that aims to identify unjust and oppressive forces. Naming racism, for instance, allows us to accurately identify its perpetrators as racist in a way that subsuming it under the category of disability may not.

Is it possible that Douglas Baynton’s exhortation to historians to notice that ‘disability is everywhere in history’ has been taken too far?^37^ Part of the job of the historian is to show that the very different context of the past makes comparisons with the present useless: ‘history cannot fully work as a precedent because the system of social references that made something happen in the first instance can never be fully recreated’.^38^ Precision about the past is critical for disability historians working under the maxim ‘nothing about us without us’, for whom part of the work of disability history is to look to the future and guard the present. Without undue anachronism, some degree of disability activism is also part of the job of the disability historian. In the preface to his book, Hogan reflects on presenting his early research and being asked ‘Why have you chosen to freely interchange the terms *genetic disease* and *disability* here?’.^39^ This question pointed out that the medical model understanding of genetic disease did not overlap with a social model understanding of disability and questioned their simplistic equation. It is a question that has important ramifications for the growing number of people diagnosed as having or carrying genetic diseases and, as Hogan suggests, disability activism in this arena can benefit from ‘the tools of a historian’.^40^ This phrase remained in my mind when reflecting on these three books. Whether we are working to swell the ranks of disabled professionals in medicine, expand the geographic and chronological reach of the field or reveal the interrelationship between race and disability, what we are working towards is not (only) a stronger academic discipline but a more equitable future.

## Footnotes

1 Andrew J. Hogan, *Disability Dialogues: Advocacy, Science, and Prestige in Postwar Clinical Professions* (Baltimore: Johns Hopkins University Press, 2022), 26.

2 Hogan, *Disability Dialogues*, 26.

3 Beth Linker, ‘On the Borderland of Medical and Disability History: A Survey of the Fields’, *Bulletin of the History of Medicine* 87, no. 4 (2013): 499–535.

4 Marion Schmidt, *Eradicating Deafness? Genetics, Pathology, and Diversity in Twentieth-Century America* (Manchester: Manchester University Press, 2020); Mara Mills, Jaipreet Virdi and Sarah F. Rose, ‘Disability, Epistemology, Sciencing’, *Osiris* 39 (2024): 1–24; Coreen McGuire, ‘What Is Disability History the History of?’, *History Compass* 22, no. 6 (2024).

5 Andrew Hogan, ‘Moving Away from the “Medical Model”: The Development and Revision of the World Health Organization’s Classification of Disability’, *Bulletin of the History of Medicine* 93, no. 2 (2019): 241–69.

6 Hogan, *Disability Dialogues*, 165.

7 Hogan, *Disability Dialogues*, 5.

8 Hogan, *Disability Dialogues*, 154.

9 Hogan, *Disability Dialogues*, 135.

10 David M. Turner and Daniel Blackie, ‘Disability and Political Activism in Industrialising Britain, c. 1830–1850’, *Social History* 47, no. 2 (2022): 130.

11 Hogan, *Disability Dialogues*, 54.

12 Hogan, *Disability Dialogues*, 48.

13 Esme Cleall, ed., *Global Histories of Disability, 1700–2015: Power, Place and People* (Abingdon: Routledge, 2023), 2.

14 Cleall, *Global Histories of Disability*, 13.

15 Cleall, *Global Histories of Disability*, 13.

16 Daniel Blackie and Alexia Moncrieff, ‘State of the Field: Disability History’, *History* 107, no. 377 (2022): 789–811.

17 Iain Hutchinson, ‘Coup de Soleil – William Baillie (1789–1869) and an Eastern (Mis)Adventure’, in *Global Histories of Disability, 1700–2015: Power, Place and People*, ed. Esme Cleall (Abingdon: Routledge, 2023), 129–45.

18 Hutchinson, ‘Coup de Soleil’, 144.

19 Hutchinson, ‘Coup de Soleil’, 144.

20 Caroline Lieffers, ‘Imperial Mobilities: Disability, Indigeneity, and the United State West, 1850–1920’, in *Global Histories of Disability, 1700–2015: Power, Place and People*, ed. Esme Cleall (Abingdon: Routledge, 2023), 95.

21 Caroline Lieffers, ‘Imperial Mobilities: Disability, Indigeneity, and the United State West, 1850–1920’, in *Global Histories of Disability, 1700–2015: Power, Place and People*, ed. Esme Cleall (Abingdon: Routledge, 2023), 96.

22 Madhwi, “‘Able”, “Disabled”, and “Invalid” Labourers: Disability and Indenture in Mauritius and Natal, c.1840–1910’, in *Global Histories of Disability, 1700–2015: Power, Place and People*, ed. Esme Cleall (Abingdon: Routledge, 2023), 45.

23 Madhwi, “‘Able”, “Disabled”, and “Invalid” Labourers’, 53.

24 Madhwi, “‘Able”, “Disabled”, and “Invalid” Labourers’, 46.

25 For explanation and critique of the industrialisation thesis, see Turner and Blackie, *Disability in the Industrial Revolution*.

26 Stefanie Hunt-Kennedy, ‘The Middle Passage, the Market, and the Plantation: Slavery-Induced Disability in the Eighteenth-Century Caribbean’, in *Global Histories of Disability, 1700–2015: Power, Place and People*, ed. Esme Cleall (Abingdon: Routledge, 2023), 36.

27 Sam De Schutter, ‘The Colonial Invention of Disability: The Politics of Disability and Productivity in Kenya, c.1940–1970’, in *Global Histories of Disability, 1700–2015: Power, Place and People*, ed. Esme Cleall (Abingdon: Routledge, 2023), 57.

28 De Schutter, ‘The Colonial Invention of Disability’, 57.

29 De Schutter, ‘The Colonial Invention of Disability’, 62. See Sarah F. Rose, *No Right to be Idle: The Invention of Disability, 1840s–1930s* (Chapel Hill: University of North Carolina Press, 2017).

30 De Schutter, ‘The Colonial Invention of Disability’, 61.

31 The enduring importance of labour to our understanding of disability and the development of welfare policies is given full treatment in Radu Harald Dinu and Staffan Bengtsson’s edited collection. Not only does this book offer eleven case studies demonstrating how disabled people have been variously excluded and integrated into the labour force, it also brings a welcome focus to state-socialist countries across Scandinavia and eastern Europe. See Radu Harald Dinu and Staffan Bengtsson, *Disability and Labour in the Twentieth Century Historical and Comparative Perspectives* (Abingdon: Routledge, 2023).

32 Stefanie Hunt-Kennedy, *Between Fitness and Death: Disability and Slavery in the Caribbean* (Urbana: University of Illinois Press, 2020).

33 Hunt-Kennedy, *Between Fitness and Death*, 11.

34 Hunt-Kennedy, *Between Fitness and Death*,110.

35 Hunt-Kennedy, *Between Fitness and Death*, 163.

36 Hunt-Kennedy, *Between Fitness and Death*, 112.

37 Douglas C. Baynton, ‘Slaves, Immigrants, and Suffragists: The Uses of Disability in Citizenship Debates’, *PMLA* 120, no. 2 (2005): 562–67.

38 Anna Greenwood, *Arts for Health: History* (Leeds: Emerald Publishing, 2023), 110.

39 Hogan, *Disability Dialogues*, preface.

40 Hogan, *Disability Dialogues*, preface.

